# The Roles of Immune Cells in the Pathogenesis of Fibrosis

**DOI:** 10.3390/ijms21155203

**Published:** 2020-07-22

**Authors:** Enyu Huang, Na Peng, Fan Xiao, Dajun Hu, Xiaohui Wang, Liwei Lu

**Affiliations:** 1 Department of Pathology and Shenzhen Institute of Research and Innovation, The University of Hong Kong, Hong Kong, China; u3004516@connect.hku.hk (E.H.); xiaof@hku.hk (F.X.); 2 Department of Rheumatology and Immunology, the Second People’s Hospital of Three Gorges University, Yichang 443000, China; doctorpeng0836@163.com (N.P.); 13487210688@163.com (D.H.)

**Keywords:** fibrosis, innate immune cells, adaptive immune cells, myofibroblast, molecular mechanism

## Abstract

Tissue injury and inflammatory response trigger the development of fibrosis in various diseases. It has been recognized that both innate and adaptive immune cells are important players with multifaceted functions in fibrogenesis. The activated immune cells produce various cytokines, modulate the differentiation and functions of myofibroblasts via diverse molecular mechanisms, and regulate fibrotic development. The immune cells exhibit differential functions during different stages of fibrotic diseases. In this review, we summarized recent advances in understanding the roles of immune cells in regulating fibrotic development and immune-based therapies in different disorders and discuss the underlying molecular mechanisms with a focus on mTOR and JAK-STAT signaling pathways.

## 1. Introduction

Fibrosis is the abnormal formation of excess fibrous connective tissue during chronic inflammation and tissue repair. The excessive depositions of extracellular matrix (ECM) components especially collagens are the leading cause of fibrosis. A variety of stimuli such as allergic responses, autoimmune development, persistent infections, and tissue injury contribute to the initiation and progression of fibrogenesis in various tissues and organs including skin, liver, lung, heart, and kidney [1]. Fibrosis is a common pathway to organ injury and failure in many diseases including systemic sclerosis (SSc), idiopathic pulmonary fibrosis (IPF), and the recent pandemic Coronavirus disease 2019 [1,2]. Fibrosis is often associated with poor prognosis and accounts for substantial morbidity and mortality [1].

Lines of evidence from clinical observations and animal studies have demonstrated the involvement of various cell subsets during fibrogenesis. The activation of myofibroblasts, one of the primary producers of ECM, is the key fibrogenic event in fibrotic diseases. Furthermore, compelling evidence suggests that both innate and adaptive immune cell populations are critically involved in the regulation of myofibroblast activation and fibrogenic responses in many fibrotic diseases [3,4]. During inflammatory responses, activated immune cells orchestrate the cellular and molecular processes of fibrosis in responses to external stimuli and microenvironmental factors. The recruitment and activation of immune cells including macrophages, neutrophils, natural killer (NK) cells, T cells, and B cells regulate the progression and regression of fibrogenic development in various organs and tissues through different molecular mechanisms.

In this review, we described and summarized recent advances in the understanding of the key roles of immune cells in fibrosis pathogenesis and the underlying molecular mechanisms, which may shed new light on the development of novel anti-fibrotic strategies by modulating immune cell functions.

## 2. Innate Immune Cells in the Pathogenesis of Fibrosis

### 2.1. Macrophages

Macrophages are important regulators of tissue repair and fibrosis. It has been recognized that macrophages undergo differentiation with phenotypic and functional changes during initiation and progression of fibrosis development [5]. Macrophages are heterogeneous populations with multiple phenotypes and diverse plasticity under the influence of different microenvironmental factors in various organs [6]. Based on their differential cytokine profiles and effector functions, macrophages are divided into classically activated (M1) and alternatively activated (M2) macrophages subsets. Although this classification does not adequately describe the diverse phenotypes of macrophage subsets, previous studies have suggested an involvement of these macrophages in fibrogenesis [7]. Moreover, increasing evidence indicates that tissue resident macrophages including Kupffer cells are key players in tissue fibrosis.

Accumulated data have suggested the critical roles of macrophages in fibrogenic responses. Conditional depletion of macrophages using CD11b-DTR mice markedly decreased numbers of myofibroblasts and attenuated carbon tetrachloride (CCl_4_)-induced liver fibrosis, suggesting a pro-fibrogenic role of macrophages in the fibrotic model [8]. Consistently, macrophages contributed to NF-κB-induced liver fibrosis in a liver injury-independent manner, possibly through regulating myofibroblast survival [9,10]. Moreover, inflammatory Gr1^+^ monocytes were recruited into injured liver in a CCR2-dependent manner and differentiated into LY6C^hi^ CD11b^+^ F4/80^+^ macrophages that strongly promoted liver fibrosis [11]. As heterogeneous populations, monocytes and macrophages comprise different functional subsets. A new subset of segregated-nucleus-containing atypical monocytes was identified as a critical pro-fibrotic player in bleomycin-induced lung fibrosis [12]. In addition, an expression quantitative trait locus analysis in monocyte-derived macrophages revealed that changes of macrophage transcriptome were closely associated with the susceptibility of SSc, an autoimmune disease with fibrosis in skin and internal organs [13].

Macrophages polarize to M1 phenotypes in response to IFN-γ and TNF-α, and M2 phenotypes in response to IL-4, IL-10, IL-13, and TGF-β [14]. Previous studies have suggested that both M1 and M2 macrophages are involved in fibrosis development. M1 macrophages produce massive amounts proinflammatory cytokines and chemokines that promote tissue inflammation and myofibroblast activation and differentiation [7]. However, M1 macrophages can also release matrix metalloproteinases (MMPs) including MMP12 that may degrade ECM and contribute to the resolution of fibrosis, suggesting an anti-fibrotic role of these cells [15].

M2 macrophages exhibit anti-inflammatory properties and play a pro-fibrotic role in fibrosis. F4/80^+^CD301^+^ M2 macrophages were found to be the dominant population in kidneys of mice with unilateral ureteral obstruction (UUO)-induced renal fibrosis. Depletion of these M2 macrophages by clodronate liposome administration ameliorated renal fibrosis and reduced epithelial-to-mesenchymal transition while transfer of M2 macrophages into kidney capsules increased the expressions of α-smooth muscle actin (α-SMA), an important indicator of fibrosis [16]. Similarly, significantly increased numbers of M2 macrophages in lung tissues were detected in mice with bleomycin-induced pulmonary fibrosis and patients with IPF, which was associated with the upregulation of macrophage alternative activation signature genes [17,18]. Depletion of alveolar macrophages or circulating monocytes by clodronate liposome treatment markedly reduced lung fibrosis [17]. Moreover, mice with IL-10-induced pulmonary fibrosis showed significantly increased numbers of M2 macrophages in both bronchoalveolar lavage and lung tissue than normal mice, which was associated with enhanced expression of C-C motif chemokine ligand 2 (CCL2). Notably, anti-CCL2 antibody treatment attenuated lung fibrosis in these mice, suggesting that targeting M2 macrophages may represent a promising therapeutic strategy for treating fibrosis [19]. Alveolar M2 cells promote myofibroblast differentiation through regulating Akt1 activation and mitophagy. Phosphorylation of Akt1 was observed in the alveolar macrophages of IPF patients and bleomycin-treated mice. Akt1 increased the production of ROS and induced mitophagy. Constitutive activation of Akt1 enhanced TGF-β expression which was regulated by Park2, an important E3 ubiquitin ligase in mitophagy. Thus, the Akt1–ROS–mitophagy pathway promoted the pro-fibrotic effects of M2 macrophages [20]. 

Tissue resident macrophages have been recognized as important players in fibrosis. As major resident macrophages in liver, Kupffer cells have a crucial role in sustaining liver immune homeostasis. The numbers of Kupffer cells were decreased during hepatic inflammation and fibrogenesis [4]. Moreover, Kupffer cells produced pro-fibrotic cytokines including TGF-β and platelet-derived growth factor (PDGF) that activated hepatic stellate cells (HSCs) and promoted fibrogenic responses [21]. Kupffer cells also secreted multiple MMPs that contributed to the resolution of fibrosis, suggesting the multiple functions of Kupffer cells in liver fibrosis [22]. Although tissue resident macrophages differ significantly from monocyte-derived macrophages, a population of alveolar macrophages with tissue resident phenotypes derived from monocytes promoted lung fibrosis and persisted in the lung over the life span, suggesting that targeting tissue resident alveolar macrophage differentiation may ameliorate pulmonary fibrosis [23].

Macrophages are important players in the development of steatosis, inflammation and fibrosis in nonalcoholic fatty liver disease (NAFLD) and nonalcoholic steatohepatitis (NASH). The polarization of inflammatory monocytes and activation of adipose tissue macrophages in the visceral compartment are both critically involved in the disease pathogenesis [24]. The portal infiltration of macrophages has been identified as an early event in human NAFLD, occurring before the development of inflammation or fibrosis, which may also serve as a predictive factor for disease progression [25]. Kupffer cells respond to hepatocyte injuries at early stage and produce massive amount of TNF -α, IL-1-β, and chemokines, which promote monocyte recruitment and inflammatory process during NASH/NAFLD development [26,27]. Mice with depletion of liver Kupffer cells by gadolinium chloride were protected from the development of diet-induced hepatic steatosis and insulin resistance [28]. Moreover, M2 Kupffer cells induced M1 cell apoptosis in culture and attenuated alcohol- and high fat diet-induced fatty liver diseases in mice, suggesting that M2 macrophages play a protective role in the development of NAFLD [29].

### 2.2. Neutrophils

Neutrophils are recruited to inflammatory sites at early stages of wound healing and play an important pro-fibrotic role in fibrosis. Depletion of neutrophils significantly attenuated lung fibrosis, suggesting a critical pro-fibrotic role of neutrophils [30]. It has been shown that neutrophils are recruited to damaged lungs dependent on formyl peptide receptor 1 while failure in recruiting neutrophils or depletion of neutrophils protect mice from bleomycin-induced pulmonary fibrosis [31]. Moreover, neutrophil-secreted elastase (NE) has been shown to play a pro-fibrotic role in asbestos-induced lung fibrosis. Deficiency of NE or treatment of NE antagonists ONO-5046 resulted in significantly decreased myofibroblast cell numbers and collagen deposition in mice with asbestos-induced lung fibrosis. Moreover, endocytosis of NE by fibroblasts increased α-SMA expression, promoted cell proliferation and enhanced migration and contractility of these cells independent of PI3K/Akt activation [32]. A clinical study has reported higher levels of NE in lung parenchyma, bronchoalveolar lavage fluid (BALF) and sera from patients with IPF when compared with those in normal subjects, suggesting an involvement of NE in the pathogenesis of IPF [33]. Furthermore, pharmacological inhibition of neutrophil extracellular trap formation may represent a promising therapy for the treatment of various inflammatory diseases [34].

### 2.3. NK Cells

Numerous studies have suggested the anti-fibrotic roles of NK cells in liver fibrosis through regulating HSCs that can differentiate into myofibroblasts and contribute to collagen deposition. It was found that hepatic NK cells killed activated HSCs dependent on TNF-related apoptosis-inducing ligand (TRAIL) and NKG2D-mediated signaling in mice with CCl_4_-induced liver fibrosis [35]. IFN-γ stimulation upregulated NKG2D and TRAIL expressions and increased the cytotoxicity of hepatic NK cells [35]. NK cells can also eliminate senescent HSCs during fibrosis progression. NKG2D-deficient mice showed increased numbers of senescent cells around the fibrotic scar areas of liver with increased collagen depositions in mice with CCl_4_ challenge [36]. Consistently, curcumin increased NKG2D ligands expressions in HSCs and enhanced the cytotoxicity of NK cells from mice with CCl_4_-induced liver fibrosis, suggesting a role of NKG2D in regulating the killing capacities of NK cells [37]. Clinical observations showed that a subset of CD56^dim^KLRG1^+^ NK cells decreased in the peripheral blood and liver of patients with chronic HBV infection at advanced stages of fibrosis. These NK cells exhibited high level of IFN-γ expression and TRAIL-dependent cytotoxicity against HSCs, which could be further enhanced by IFN-γ and CD44 stimulations [38].

NK cells have been shown to suppress cardiac fibrosis in experimental autoimmune myocarditis. Eosinophil infiltration and collagen deposition were increased in the heart of mice with cardiac fibrosis. However, depletion of NK cells exacerbated cardiac fibrosis, which was dependent on eosinophils. Moreover, NK cells induced the apoptosis of eosinophils in culture, suggesting that NK cells play a protective role in cardiac fibrosis by regulating eosinophils [39].

NK cells also exhibited pro-fibrotic roles in some conditions. Depletion of NK cells ameliorated leukocyte infiltration and liver fibrosis in a low-dose rotavirus model [40]. Moreover, CD161^+^CD3^−^ NK cells secreted massive amounts of IL-17 and promoted acute kidney injury-induced renal fibrosis in athymic mice while blockade of IL-17 attenuated renal inflammation and fibrosis, suggesting that these NK cells contributed to renal fibrosis by producing IL-17 [41].

### 2.4. Innate Lymphoid Cells

Innate lymphoid cells (ILCs) are heterogeneous innate immune populations that lack antigen-specific receptors and lineage markers, which play important roles in sustaining immune homeostasis and innate immune responses against pathogens. ILCs are mainly classified into three groups based on their signature cytokines and transcription factors. Indeed, three groups of ILCs, namely ILC1, ILC2, and ILC3, mirror the cytokine signatures of Th1, Th2, and Th17 cells, respectively [42]. Recent evidence indicates that ILCs exert significant functions in tissue repair and fibrosis development [43,44].

Clinical observations have shown that an ILC2 population was detected in the lungs of IPF patients [45]. Moreover, the percentages of CD69^+^ ILC2s were positively correlated with aggravation of liver fibrosis in patients with liver diseases, suggesting that ILC2s may contribute to the immunopathology of liver fibrosis [46]. The pro-fibrotic roles of ILC2 in pulmonary fibrosis were further supported by animal studies. IL-13 produced by IL-25-activated ILC2s was shown to drive collagen deposition in the lungs of mice with bleomycin-induced fibrosis [45]. Intranasal delivery of recombinant IL-25 also induced pulmonary inflammation and fibrosis, which was associated with connective tissue growth factor and TGF-β production [45]. Moreover, IL-33, a cytokine closely associated with organ fibrosis, also promoted IL-13 production by ILC2s, suggesting that IL-33 might drive fibrosis via regulating ILC2 functions [47]. 

ILC1s and ILC3s are also involved in organ fibrosis pathogenesis. It was recently reported that ILC1s accumulated in adipose tissues of patients with type 2 diabetes whereas these cells promoted adipose fibrogenesis through activation of TGF-β signaling [48]. Furthermore, inhibition of adipose accumulation of ILC1s attenuated adipose tissue fibrosis, suggesting that ILC1s might be promising therapeutic targets for treating adipose fibrosis [48]. ILC3s are characterized by the production of IL-17, which exhibited elevated levels in patients with IPF and SSc [44]. The frequencies of ILC3s with IL-17 production were increased in liver from CCl_4_-induced fibrotic mice than those from normal mice. Adoptive transfer of ILC3s into ILC-depleted mice increased ECM accumulation and promoted liver fibrosis, suggesting a pro-fibrotic role of ILC3s in liver fibrotic progression [49].

### 2.5. γδT Cells

γδT cells showed diverse functions during fibrosis development. It was reported that adoptively transferred γδT cells from CCl_4_-treated mice infiltrated into liver and reduced hepatic inflammation and fibrosis through inducing apoptosis of HSCs in recipient mice, suggesting a protective role of γδT cells in liver fibrosis [50]. A subset of Vγ6Vδ1 γδT cells actively secreted IL-17 and dramatically increased in mice with repeated *B. subtilis* infection. These Vγ6Vδ1 γδT cells were shown to be anti-fibrotic, because Vγ4/6 or δ chain deficiency led to severer collagen deposition while Vγ6Vδ1 transgenic mice exhibited alleviated pulmonary fibrosis [51]. Moreover, Vγ6 γδT cells and a subset of γδTCR^lo^ γδT cells expressed IL-22 in an aryl hydrocarbon receptor (AhR)-dependent manner. Deficiency of γδTCR or inhibition of AhR signaling aggravated lung fibrosis, which was restored by nasal inhalation of recombinant IL-22 [52]. A subset of NK1.1^+^ γδT cells suppressed fibrosis in bleomycin model via IFN-γ secretion. Transfer of NK1.1^+^ γδT cells to δ chain deficient mice partially ameliorated lung fibrosis [53]. In contrast, depletion of Vγ2 γδT cells decreased collagen fiber in liver of mice with *S. japonicum* infection-induced liver fibrosis, suggesting a pro-fibrotic role of these cells [54].

### 2.6. Dendritic Cells

Growing evidence indicates that dendritic cells (DCs) are novel players in the pathogenesis of various fibrotic diseases [55,56]. DCs are potent antigen-presenting cells with key roles in modulating immune responses. Recent studies have revealed the involvement of different DCs subsets in the development of fibrosis. The frequencies of circulating conventional CD141^+^ and CD1c^+^ DCs, namely cDC1 and cDC2, and CD303^+^ plasmacytoid DCs (pDCs) were significantly reduced in patients with IPF when compared with those in age and sex matched healthy controls [57]. Moreover, a subset of BDCA1^+^ DCs were detected in the lungs of patients with IPF or hypersensitivity pneumonitis, suggesting a potential role of BDCA1^+^ DCs in lung fibrosis [58]. Consistently, accumulated DCs in lung tissue were observed in mice with pulmonary fibrosis. Selective depletion of lung DCs markedly exacerbated lung fibrosis in mice, suggesting a protective role of lung DCs in fibrogenesis [59]. Furthermore, increased mobilization of lung CD11b^+^ DC regulated pulmonary fibrosis development in mice [60]. These studies have suggested the potential of DC-based immunotherapy for the treatment of lung fibrosis.

Increasing evidence indicates that DCs are involved in cardiac fibrosis. The infiltrated CD209^+^ DCs and CD11c^+^ DCs in human infarcted heart were increased in patients with cardiac rupture, which were associated with impaired cardiac reparative fibrosis [61]. Moreover, the CD11b^+^CD11c^+^ tolerogenic DCs with low expression of MHC-II, CD86, CD80 and high level of IL-10 production reduced heart inflammation and fibrosis in a mouse model of chronic Chagas disease cardiomyopathy [62]. The protective roles of tolerogenic DCs in cardiac fibrosis appear to be associated with reduced expressions of pro-inflammatory cytokines and increased IL-10 production [62]. Recent studies have suggested that pDCs are involved in SSc pathogenesis [56]. The pDCs infiltrated into the skin of SSc patients and produced large amounts of CXCL4 and IFN-α [63]. CXCL4 and DNA formed liquid crystalline complexes and activated pDCs in a TLR-9-dependent manner, which promoted IFN-α production by pDCs [63]. Depletion of pDCs attenuated fibrosis of the skin and lung in the bleomycin-induced SSc mice, indicating a pathogenic role of pDCs in SSc pathogenesis [64]. A group of classical CD11b^+^ DCs played a profibrotic role in a mouse model of allergic eye disease (AED), which was dependent on activation of the retinoic acid pathway [65]. The classical CD11b^+^ DCs within ocular mucosa exhibited activation of aldehyde dehydrogenase (ALDH), a critical enzyme required for retinoic acid synthesis. The DCs-derived ALDH increased ligation of retinoic acid with conjunctival fibroblast retinoid X receptor (RXR) and induced rapid onset of ocular mucosal fibrosis [65].

### 2.7. NKT Cells and Mucosal-Associated Iinvariant T (MAIT) Cells

Recent studies have revealed a role of NKT cells in the development of fibrosis. In HBV-transgenic mice that resemble human HBV carriers, CCl_4_-induced liver fibrosis becomes more pronounced than that in wild type mice. Depletion of NK cells and NKT cells or blockade of CD1d reduces the levels of α-SMA expression in the liver, while depletion of NK cells alone shows no such effect. Moreover, blockade of IL-4 or IL-13 inhibits the effects of NKT cells on upregulating α-SMA in HSCs in vitro, suggesting that NKT cells promote liver fibrosis via Th2 cytokines in HBV-associated liver fibrosis [66]. In a diet-induced NAFLD mouse model, *CD1d*^−/−^ mice showed severer T cell infiltration in the kidneys with increased renal expression of TLR4, TGF-β, and α-SMA. However, the renal pathology was ameliorated in TLR4-deficient mice, suggesting that CD1d-dependent NKT cells played a protective role in NAFLD-associated renal inflammation and fibrosis via suppressing TLR4-mediated signaling function [67]. The *Rag2*^−/−^ mice overexpressing TCR Vα3.2 and Vβ9 chains showed increased generation of type II NKT cells and spontaneously developed hepatitis and liver fibrosis, in which type II NKT cells produced sufficient Th2 cytokines and contributed to the liver fibrosis [68].

Invariant NKT cells (iNKT) play a protective role in bleomycin-induced pulmonary fibrosis via secretion of IFN-γ. The increase of TGF-β and collagen deposition in the lung tissue of *CD1d*^−/−^ diseased mice could be reversed by transfer of iNKT cells from wild type mice but not IFN-γ-deficient mice [69]. However, repeated intranasal administration of α-Galactosylceramide (α-Galcer) induced chronic obstructive pulmonary disease (COPD)-like symptoms in mice, with increased leukocyte infiltration in the lung tissues and fibrosis of airways. These COPD-like symptoms could be reversed by blockade of IL-4, suggesting that iNKT cells promoted airway inflammation and fibrosis via IL-4 [70]. In 2-OA-BSA-induced autoimmune cholangitis mice, activation of iNKT cells by α-Galcer treatment promoted lymphocyte infiltration and collagen deposition in the liver, which was ameliorated in CD1-deficient mice, indicating a profibrotic role of iNKT cells in primary biliary cholangitis (PBC) [71]. An early study of NASH using mice fed with high fat diet showed that Jα18-deficient mice with iNKT cell deficiency exhibited severer hepatic inflammation and ultimately extensive liver fibrosis, suggesting a protective role of iNKT cells in NASH [72]. However, NASH and liver fibrosis were ameliorated in the liver of CD1d-deficient mice fed with high-fat high-carbohydrate diet, suggesting that CD1d-dependent NKT cells were profibrotic in NASH [73]. Given that CD1d-dependent NKT cells comprise various subsets including iNKT cells, the different subsets may play opposite roles in the pathogenesis of NASH [74].

Mucosal-associated invariant T cells (MAIT cells) are decreased in the liver and blood of patients with autoimmune liver diseases (AILDs). MAIT cells could induce HSC proliferation and expression of collagen and proinflammatory cytokines in vitro [75]. Jiang et al. found decreased numbers of circulating MAIT cells in patients with PBC, a subset of AILDs. Activated MAIT cells promoted hepatic myofibroblast (HMF) proliferation in an MR-1 dependent manner, and induced IL-6 and IL-8 expression in HMF. CCl_4_-induced liver fibrosis was decreased in *MR1*^−/−^ mice, while both CCl_4_-induced and bile duct ligation-induced liver fibrosis were exacerbated in Vα19 TCR transgenic mice with enhanced MAIT cell generation, demonstrating a profibrotic role of MAIT cells in liver fibrosis [76]. In patients with chronic HCV-infection, the liver hepatic inflammation and fibrosis scores were negatively correlated with the frequencies of MAIT cells in liver, suggesting that MAIT cells may be involved in HCV-associated liver fibrosis [77]. A study on chronic kidney disease showed that the numbers of MAIT cells in the tissues were positively correlated with the severity of renal fibrosis. MAIT cells were in close proximity with proximal tubular epithelial cells (PTECs) in the fibrotic kidneys, suggesting that MAIT cells may contribute to fibrogenesis via targeting PTECs in chronic kidney disease [78].

## 3. Adaptive Immune Cells in the Pathogenesis of Fibrosis

### 3.1. Th1 Cells

Th1 cells and their secreted cytokines including IFN-γ and IL-12 have been suggested to exert anti-fibrotic functions under various conditions. Administration of IL-12, a potent inducer of Th1 cells, attenuated bleomycin-induced pulmonary fibrosis through inducing IFN-γ production, suggesting a protective role of Th1-associated cytokines in fibrosis [79]. Furthermore, deficiency of T-bet, the signature transcription factor of Th1 cells, resulted in markedly increased expression of the profibrotic factor TGF-β and deposition of collagen in lungs from bleomycin-treated mice [80].

However, recent findings have revealed pro-fibrotic roles of Th1 cells and associated cytokines in fibrosis. *S. epidermidis* triggered IL-6-dependent Th1 expansion which activated STAT1 in peritoneal membrane and subsequent peritoneal fibrosis. IFN-γ deficiency resulted in significantly ameliorated *S. epidermidis-*induced peritoneal fibrosis while adoptive transfer of Th1 cells induced fibrotic progression in fibrosis-resistant IL-6 deficient mice [81]. Similarly, it was shown that Th1 cells induced TGF-β production in cardiac fibroblasts and selectively drove cardiac fibrosis in an IFNγ-dependent manner, suggesting a pro-fibrotic role of Th1 cells and IFN-γ in nonischemic heart failure [82].

### 3.2. Th2 Cells

Extensive evidence suggests that type 2 immunity contributes to the development of fibrosis in different organs [83]. The important roles of IL-4 and Th2 cells in bacterial infection-associated hepatic fibrosis have long been recognized [84]. The pro-fibrotic roles of Th2 cells were also demonstrated in pulmonary fibrosis. It has been shown that chronic asthma induces the thickening and fibrosis of bronchial basement membrane. Administration of house dust mites in the airways induced amphiregulin-producing memory Th2 cells, which further enhanced airway inflammation-induced fibrosis [85]. The pathogenic memory Th2 cells-derived amphiregulin promoted myofibroblast differentiation and sub-basement membrane fibrosis of the airway through the induction of osteopontin by infiltrated inflammatory eosinophils [85]. The pathogenic roles of amphiregulin-producing memory Th2 cells may be potential targets for the treatment of fibrosis induced by chronic allergic disorders.

### 3.3. Th17 Cells

In addition to their roles in immunity and inflammation, Th17 cells are actively involved in fibrogenic responses with different roles in various fibrotic diseases. Th17 cells produce massive amounts of IL-17 and IL-22, both of which are associated with tissue fibrosis. IPF patients showed increased levels of IL-17 in BALF than normal volunteers [86]. Animal studies indicated that Th17 cells and IL-17 promoted skin and lung fibrosis development in a bleomycin-induced murine model of SSc [87]. It has been reported that IL-17 exhibits dual roles in pulmonary fibrosis. Mice with IL-17RA deficiency showed impaired clearance of *B. subtilis* and increased lung fibrosis, suggesting a protective role of IL-17 [51]. However, IL-17 deficient mice exhibited attenuated bleomycin-induced pulmonary fibrosis [86]. Moreover, intratracheal administration of IL-17 was shown to induce collagen accumulation and fibrotic lesions while neutralization of IL-17 reduced tissue fibrosis, indicating a pro-fibrotic role of IL-17 in chemical-induced fibrosis [86]. The frequencies of circulating Th17 cells were increased in patients with HBV infection-induced liver cirrhosis [88]. Moreover, Th17 frequencies were higher in liver tissues with more advanced fibrosis [89,90], suggesting a role of Th17 cells in the development of liver fibrosis.

Increased hepatic Th17 cells were detected in patients with advanced-stage HBV-related liver fibrosis [90]. Animal studies suggested a pro-fibrotic role of IL-17 in liver injury-induced fibrosis. IL-17 signaling facilitated the production of multiple cytokines including TGF-β, and directly induced production of collagen in HSCs through activation of signal transducers and activators of transcription (STAT) 3 signaling pathway [91]. Inhibition of Th17 cells by small molecules Halofugine and Magnolol significantly reduced the severity of Concanavalin A-induced liver fibrosis, suggesting that Th17 cells may represent novel therapeutic targets for treating liver fibrosis [92,93]. 

IL-22 has been shown to suppress pulmonary fibrosis. Bleomycin-treated mice exhibited markedly reduced levels of IL-22 in the lung [94]. Intraperitoneal injection of anti-IL-22 antibodies upregulated fibrosis-associated molecules including α-SMA, collagens and TGF-β in lung tissues of mice with bleomycin-induced fibrosis [94]. However, it has been suggested that IL-22 may contribute to HCV-associated liver fibrosis. Increased intrahepatic IL-22-producing cells were positively correlated with fibrotic severities in patients with HCV infection while IL-22 increased α-SMA expression and collagen production by HSCs in culture [95]. Thus, current data indicate a dual role of IL-22 in fibrosis development.

### 3.4. Regulatory T Cells

Increasing evidence indicates a pivotal role of regulatory T cells (Tregs) in fibrogenic responses. An imbalance between Tregs and Th17 cells was observed in IPF patients while the frequencies of Tregs were negatively correlated with the severities of IPF [96,97]. Moreover, depletion of Tregs by anti-CD25 antibodies at a late stage increased fibrotic scores and hydroxyproline content in the lungs of bleomycin-treated mice, suggesting a protective role of Tregs in pulmonary fibrosis [98]. Similarly, Tregs played a protective role against pneumococcus-induced lung fibrosis in mice [99]. Depletion of Tregs by diphtheria toxin resulted in increased lung collagen deposition, elevated Th1/Th2 cytokine levels and exacerbated infection-induced pulmonary fibrosis [99]. However, Tregs expansion markedly attenuated pneumococcus-induced fibrosis in mice [99]. Tregs suppressed TGF-β-induced pulmonary fibrosis through decreasing fibroblast growth factor 9 (FGF-9) expression by parenchymal cells and alveolar macrophages [100]. Depletion of Tregs in vivo increased inflammatory cytokine production and exacerbated hepatic fibrosis in mice with bile duct ligation (BDL) [101]. Moreover, Treg depletion resulted in increased Th2 cytokine production and skin fibroblast activation with upregulated profibrotic gene expressions in a bleomycin-induced murine model of skin sclerosis [102]. The conditional deletion of *Gata3* in Tregs led to increased fibroblast activation and dermal fibrosis, suggesting an important role of *Gata3* in modulating Tregs function during fibrosis development [102]. A recent study also revealed a protective role of Tregs in kidney injury and fibrosis [103].

It has been shown that the mammalian target of rapamycin (mTOR) signaling is critically involved in regulating the protective function of Tregs [104]. Adoptive transfer of rapamycin-treated Tregs ameliorated kidney fibrosis and improved renal functions in mice with acute kidney injury, suggesting an important role of mTOR signaling in regulating Treg functions [104]. Previous studies showed that activation of AhR signal preferentially promoted gut Tregs with enhanced suppressive activities in vivo [105]. A natural AhR agonist norisoboldine promoted Tregs differentiation through regulating glycolysis and NAD+/SIRT1/SUV39H1/H3K9me3 signaling pathway during colitis development [106]. A recent study indicated a role of AhR signal in regulating Tregs functions during fibrogenic responses [107]. Bleomycin-challenged mice with treatment of FICZ, a natural AhR ligand, exhibited increased number of Tregs with attenuated lung fibrosis, suggesting a therapeutic potential of targeting AhR for treating fibrotic diseases [107]. Tregs also exerted protective functions against coxsackievirus B3-induced cardiac fibrosis via secretion of IL-10, an important regulatory cytokine [108]. IL-10 activated STAT3 signaling, inhibited p38 mitogen-activated protein kinase (MAPK) activation and suppressed HuR expression, resulting in attenuated ventricular remodeling [109]. Moreover, IL-10 suppressed HuR transcription, inhibited renin-angiotensin-aldosterone system and reduced renal fibrosis in the UUO-induced fibrosis model [110]. Notably, IL-10 exerted an inhibitory function in fibrosis by activating PI3K/AKT and STAT3 signaling pathways which were downstream mediators of the IL-10 receptor in scar-forming fibroblasts [111]. However, depletion of Tregs at early stages reduced TGF-β expression, collagen depositions and fibrosis scores in lungs of bleomycin-treated mice, indicating that Tregs may promote fibrosis at early stages of disease progression [98]. Consistently, adoptive transfer of Tregs into *Rag1*^−/−^ mice before intratracheal treatment of bleomycin exacerbated pulmonary fibrosis and increased mortality [112]. Moreover, substantial upregulation of IL-8 expression by Tregs was detected in patients with chronic hepatitis C viral infection, whereas the Tregs increased expressions of pro-fibrogenic markers by primary human HSCs [113]. Furthermore, Tregs isolated from the affected skin of SSc patients produced massive amounts of profibrotic cytokines including IL-4 and IL-13. The skin-localized Tregs expressed ST2 chain of the IL-33 receptor. IL-33, a cytokine abundantly produced in skin of SSc patients, skewed the differentiation of Treg cells into Th2-like cell phenotypes and contributed to tissue fibrosis [114].

In summary, available results have suggested the complex functions of Tregs in fibrogenic responses. Tregs may exert either protective or pathogenic roles at different stages of fibrosis development.

### 3.5. Follicular Helper T Cells

Follicular helper T (Tfh) cells are specialized CD4 helper T cells that provide help to B cells in germinal center reactions. Tfh cells produce a signature cytokine IL-21 that is critical for sustained B cell responses. It was reported that a Tfh-like cell subset infiltrated the skin of SSc patients and was associated with dermal fibrosis [115]. Moreover, an ICOS^+^ Tfh-like cell subset contributed to dermal fibrosis via producing IL-21 in the skin of graft-versus-host disease (GVHD)-SSc mice [115]. Either anti-ICOS treatment or IL-21 neutralization in GVHD-SSc mice inhibited inflammation and dermal fibrosis, suggesting that inhibition of ICOS and IL-21 might have therapeutic benefits for the treatment of SSc [115]. The proliferation and activation of Tfh cells were observed in patients with IPF [116]. It was shown that IL-21 contributed to pulmonary fibrosis through promoting the differentiation of naïve CD8 cell into pro-fibrotic CD8 T Cells in bleomycin-treated mice [117]. Both IL-21 deficient and IL-21 receptor deficient mice developed pulmonary inflammation but no fibrosis upon bleomycin challenge, suggesting an important role of IL-21 in fibrogenic development [117]. Since IL-21 is produced by various T cell subsets including Tfh cells and Th17 cells [118], the available data did not show the direct participation of Tfh cells in fibrosis. Thus, further studies are needed to delineate a role of Tfh cells in the development of fibrosis.

### 3.6. B Cells

B cells actively participate in tissue fibrosis among different organs. It was reported that deficiency of CD19 resulted in diminished B cell responses and significantly reduced susceptibility to bleomycin-induced lung fibrosis. In contrast, mice with CD19 overexpression exhibited exacerbated fibrosis, suggesting a profibrotic role of B cells in the development of pulmonary fibrosis [119]. It was suggested that B cells may promote fibrosis through regulating cytokines expression in a hyaluronan-TLR4-dependent manner [120]. Moreover, B cells were shown to promote skin fibrosis in an SSc model. B cells in TSK/+ mice that resemble human SSc symptoms showed lower stimulation thresholds with constitutive phosphorylation of CD19, increased Ca^2+^ release upon anti-CD19 activation, and enhanced IL-6 and IgG productions. CD19 deficiency inhibited B cell functions and attenuated skin fibrosis in TSK/+ mice [121]. Further studies suggested that the hyper-reactive B cell phenotypes in TSK/+ mice may partially result from dysregulated CD22 functions [122]. Moreover, deficiency of B cells markedly attenuated CCl_4_-induced fibrotic development in mice [123]. B cell-specific IL-6 deficient mice showed attenuated skin and lung fibrosis upon bleomycin challenge, suggesting a pathogenic role of B cell-derived IL-6 in the scleroderma model [124]. In culture, IL-6-producing effector B cells promoted collagen secretion by fibroblasts in a cell-cell contact-dependent manner. Notably, inhibition of B-cell activating factor (BAFF) ameliorated skin and lung fibrosis with a reduction of the effector B cells, suggesting a potential therapeutic strategy by targeting BAFF and B cells [124]. Clinical observations also suggested a role of CD19 in SSc. A case-control association study showed that functional CD19 polymorphism was associated with the susceptibility to SSc [125]. In co-culture experiments, B cells induced α-SMA and collagen expression by human dermal fibroblasts, which was further enhanced by BAFF [126]. Importantly, B cell depletion therapy with anti-CD20 treatment was well tolerated and ameliorated clinical symptoms of SSc patients [127,128].

Regulatory B cells (Bregs) exert immunosuppressive functions with secretion of anti-inflammatory cytokines, which are shown to participate in the pathogenesis of various diseases. A protective role of Bregs was supported by the evidence that B cell-specific IL-10-deficient mice exhibited significantly increased dermal thickness and exacerbated lung fibrosis in a bleomycin-induced scleroderma model [124]. However, anti-CD22 treatment preferentially depleted Bregs and attenuated lung fibrosis in mice with silica instillation-induced pulmonary fibrosis, suggesting that Bregs may promote pulmonary fibrosis [129]. These contradictory results might be attributed to different models and experimental methods for fibrosis induction. Recent evidence indicates that plasma cells possess immunomodulatory functions via IL-10 production [130]. Further studies may provide new insight in understanding the role of IL-10-producing plasma cells during the development of fibrosis in chronic autoimmune diseases [131].

## 4. The Molecular Mechanisms of Immune-Mediated Signaling Pathways in Fibrosis

### 4.1. The Roles of mTOR Signaling Pathway in Fibrosis

The mTOR protein is a key regulator of many cellular activities including proliferation, metabolism and protein synthesis. The mTOR interacts with adaptor proteins to form mTOR complex 1 (mTORC1) and mTOR complex 2 (mTORC2), both of which are involved in the pathogenesis of fibrosis. 

A number of stimuli including cytokines, growth factors and mitogens activate mTOR signaling. The activation of PI3K/AKT suppresses Tuberous Sclerosis Complex (TSC), a major negative regulator of mTORC through the GTP binding protein Ras homolog enriched in brain (Rheb). It has been shown that dysregulated mTOR signaling is closely associated with pulmonary fibrosis, liver fibrosis and SSc. Genome-wide association study suggested that mTOR signaling was associated with susceptibility to IPF [132]. The overactivation of mTOR in mesenchymal cells by conditional deletion of TSC1 exacerbated CCl_4_-induced liver fibrosis, which was reversed by mTOR inhibitor rapamycin [133]. Moreover, inhibition of mTOR by rapamycin significantly reduced the expression of pro-inflammatory cytokines and fibrogenic mediators including IL-4, IL-6, IL-17, and TGF-β, which finally resulted in attenuated skin fibrosis in both TSK/+ and bleomycin-induced SSc model mice, suggesting a pivotal role of mTOR signaling in promoting fibrosis development [134]. 

The mTOR signaling is critically involved in regulating the functions of immune cells, including DCs, macrophages, NK cells and T cells, which are key players in fibrosis pathogenesis. Inhibition of mTOR promoted IL-12 but suppressed IL-10, TNF, and IL-6 production, suggesting a proinflammatory role of mTOR in regulating immune responses [135]. It has been well-recognized that mTOR signaling regulates Tregs differentiation and functions. The protective roles of Tregs were regulated by mTOR in the repair of acute kidney injury [104]. Moreover, deletion of mTORC1 activity in CD4^+^ T cells resulted in increased inflammation, accelerated fibrosis development and increased mortality, which was associated with IL-17 production derived from γδT cells [136]. 

TGF-β, a central mediator of fibrogenesis, interacts with TGF-β receptor on fibroblasts and activates Smad proteins, which finally regulates the genes associated with EMT and fibroblast transdifferentiation [137]. TGF-β-activated mTORC1/4E-BP1 signaling through Smad phosphorylation was critical for collagen production in lung fibroblasts derived from IPF patients. Inhibition of TGF-β-induced PI3K/Akt activation showed undetectable effects on collagen production while mTOR inhibition and mTORC1 deficiency markedly reduced collagen I deposition in primary human lung fibroblasts, suggesting a role of PI3K/Akt independent activation of mTOR signaling in fibrogenesis [138]. Further analysis suggested that mTORC1/4E-BP1 axis represented a common fibrogenic signaling pathway during the development of fibrosis in different organs including lung, liver and skin [138]. Moreover, it was shown that Smad-independent TGF-β signaling also contributed to fibrosis pathogenesis by modulating p38, ERK, MAPK, and mTOR signaling [137]. TGF-β-mediated PI3K/AKT signaling may further induce activation of mTOR and subsequent fibrosis-related gene expressions [139].

### 4.2. The Roles of JAK-STAT Signaling Pathway in Fibrosis

Increasing evidence indicates that the JAK-STAT signaling pathway activation is involved in the development of many human diseases including fibrotic disorders. The binding of extracellular ligands such as cytokines, growth factors and hormones to their respective receptors activates JAKs including JAK1, JAK2, JAK3, and TYK2. Activated JAKs add phosphates at specific tyrosine residues of the receptors which serve as docking sites for the STATs [140]. The subsequent phosphorylation, dimerization and translocation of STATs regulate downstream gene expressions. Up to date, seven mammalian STAT family members have been identified, namely STAT1, STAT2, STAT3, STAT4, STAT5 (STAT5A and STAT5B), and STAT6. Recent studies have revealed that activation of the JAK-STAT signaling pathway by multiple cytokines including IL-6, IL-17, and IFNs exerts important functions in fibrosis pathogenesis.

Activated JAK-STAT signaling has been detected in various fibrotic diseases. The SSc patients showed significantly elevated phosphorylation levels of JAK1/JAK2/JAK3 and STAT3 in both skin and lung tissues when compared with healthy controls. Furthermore, inhibition of JAK by tofacitinib markedly ameliorated fibrosis development in both bleomycin-induced SSc mice and TSK1/+ mice, suggesting a critical role of JAK-STAT signaling in fibrotic changes of SSc [141]. Likewise, IPF patients exhibited enhanced levels of phosphorylated JAK2-STAT3 in lung tissues while dual inhibition of phosphorylated JAK2-STAT3 reduced lung fibrosis in mice with bleomycin challenge [142]. Moreover, both JAK2 inhibitor and STAT3 inhibitor attenuated left-atrial fibrosis in an atrial fibrillation model, suggesting a therapeutic potential of targeting JAK-STAT pathway in treating fibrosis [143].

Many cytokines derived from immune cells can activate JAK-STAT signaling pathway, which contribute to fibrosis development through various mechanisms. Type I and type II IFNs, mainly produced by pDCs, macrophages and T cells, strongly activate STAT1 phosphorylation. IFN-γ induced Smad7 expression and impaired TGF-β signaling through activating STAT1 in activated HSCs, indicating anti-fibrotic roles of IFN-γ in liver fibrosis [144]. Moreover, IFN-α reduced collagen expression and suppressed liver fibrosis though regulating STAT1 and p300 [145]. Recent evidence suggested that STAT1 activation prevented renal fibrosis by regulating macrophages differentiation and renal infiltration upon chronic kidney injury, indicating a protective role of STAT1 in renal fibrosis [146]. However, forkhead box O1, a prominent member of the forkhead box family, suppressed STAT1 activation and inhibited tubulointerstitial fibrosis in mice with diabetic kidney disease [147]. The oxidative hepatic environment in obesity increased STAT1 activation through suppressing T cell protein tyrosine phosphatase (TCPTP), which promoted hepatic fibrosis. Consistently, inhibition of the enhanced STAT1 signaling prevented T cell infiltration and liver fibrosis, suggesting a dual role of STAT1 in fibrotic development [148]. STAT3 has been suggested to integrate several profibrotic signals and serves as a core mediator of fibrosis [149]. Fibroblast-specific deficiency of STAT3 resulted in ameliorated TBRact-induced skin fibrosis in mice [149]. The JAK, JNK, SRC, and c-ABL kinases jointly activated STAT3 phosphorylation in fibroblasts with TGF-β stimulation [149]. SHP2 is an important regulator of TGF-β-induced STAT3 activation. TGF-β promoted recruitment of SHP2 to JAK2 in fibroblasts, which resulted in subsequent activation of STAT3 [150]. The inactivation of SHP2 reduced JAK2-STAT3 signaling and ameliorated dermal and pulmonary fibrosis in mice, suggesting that SHP2 and STAT3 might be molecular checkpoints for tissue fibrosis [149,150]. IL-6 is produced by epithelial cells and activated innate and adaptive immune cells including DCs, macrophages and T cells. IL-6 serves as an important activator of STAT3 and is critically involved in fibrosis. IL-6 was shown to enhance TGF-β/Smad3 signaling and collagen production, which was dependent on STAT3 activation [151]. Blocking IL-6 trans-signaling protected against kidney fibrosis by suppressing STAT3 activation in the UUO-induced renal fibrosis model [152]. Rilpivirine, a widely used anti-HIV drug, has been shown to ameliorate liver fibrosis though suppressing STAT3 and promoting STAT1-mediated HSC apoptosis [153]. Moreover, propylene glycol alginate sodium sulfate, a natural extract from brown algae, significantly reduced hepatic injury and fibrosis partially through suppressing JAK2-STAT3 activation [154].

It has been well recognized that STAT4 is activated in immune cells in responses to IL-12 and type I IFNs. The interaction of IL-12 and its receptor activates JAK2-TYK2 and STAT4, which regulates the expression of downstream cytokines such as IFN-γ. STAT4 plays essential roles in Th1 cells differentiation and functions [155]. It has been shown that STAT4 is a genetic risk factor for SSc and SSc related fibrosing alveolitis [156,157]. STAT4 deficient mice were protected against dermal fibrosis upon bleomycin challenge. These mice with STAT4 deficiency showed significantly decreased T cells infiltration and cytokines production in skin lesions, suggesting that STAT4 exerted pro-fibrotic functions by modulating T cell responses [158]. Moreover, a STAT4 variant was shown to be associated with increased hepatic inflammation and fibrosis, which might be partially attributed to the aberrant STAT4-dependent IFN-γ production by NK cells [159]. IL-12-induced STAT4 activation also contributed to cigarette smoke-induced airway fibrosis though regulating fibroblasts [160]. However, it has also been noted that IL-12 and IFN-γ show anti-fibrotic effects in bleomycin-induced pulmonary fibrosis, suggesting possible dual roles of STAT4 in regulating fibrotic diseases [79]. STAT5 proteins are activated by growth factors and cytokines. Loss of hepatic STAT5 increased TGF-β and STAT3 activation in mice upon CCl_4_ treatment, which resulted in exacerbated liver fibrosis [161]. STAT5 could directly bind to TGF-β through its N-terminal sequences and decreased TGF-β protein stability, suggesting a role of STAT5-TGF-β-STAT3 axis in liver fibrosis [161]. STAT6 is primarily activated by Th2 cytokines, including IL-13 and IL-4. IL-13 increased collagen production by activating STAT6 and promoted *S. mansoni* infection-induced liver fibrosis in a TGF-β-independent manner [162,163]. IL-4 also played a profibrotic role because deficiency of IL-4Rα signaling suppressed inflammatory monocyte infiltration and liver fibrogenesis, which was associated with reduced MMPs expression by macrophages through IL-4 and IL-13-mediated STAT6 activation [164].

## 5. Current Progress on Immune-Based Anti-Fibrotic Therapies

Recent findings on the critical involvement of immune cells in fibrogenesis have facilitated the development of novel therapeutic strategies for treating fibrotic diseases. Both pre-clinical and clinical investigations have shown a promising therapeutic potential by modulating immune cell differentiation and function for the treatment of fibrosis.

Pentraxin 2, also known as serum amyloid P, is a potent inhibitor of macrophage activation and differentiation [165]. Patients with IPF showed significantly reduced plasma levels of pentraxin 2, which was correlated with disease severities [166]. Recent studies have reported that a recombinant human pentraxin 2, named PRM-151, is well tolerated and slows the decline of lung function in IPF patients, suggesting that modulation of macrophages differentiation and activation may serve as potential therapies for treating lung fibrosis in the future [167,168].

Kupffer cells, a major resident macrophage population in liver, produce pro-fibrotic factors and contribute to liver fibrosis. Current studies have suggested therapeutic benefits by suppressing the activation of Kupffer cells [169,170]. It has been shown that TLR4-dependent activation of Kupffer cells is reduced by broad-spectrum antibiotics, which is associated with the reduction of liver fibrosis progression [169,171]. Selonsertib, an inhibitor of apoptosis signal-regulating kinase 1 (ASK1), has been shown to modulate the activation of macrophages including Kupffer cells. Pre-treatment of selonsertib reduced TNF-α expression and suppressed inflammasome activation in isolated Kupffer cells [172]. In a phase 2 clinical trial, selonsertib reduced liver fibrosis in a substantial proportion of patients with nonalcoholic steatohepatitis and stage 2–3 fibrosis, which was associated with reductions in liver stiffness, collagen content and lobular inflammation [173]. Thus, therapeutic intervention through modulating Kupffer cell activation may represent a potential therapeutic strategy for treating liver fibrosis [174].

Monocyte-derived macrophages are recruited by Kupffer cells into the liver, which promote tissue inflammation and fibrosis. Inhibition of inflammatory monocyte recruitment into liver by targeting chemokines or chemokine receptors has shown promising therapeutic benefits in various liver diseases, including liver fibrosis [175]. The serum and hepatic C-C motif chemokine ligand (CCL) 5 levels were increased in drug-induced liver injury (DILI) patients. Moreover, inhibition of CCL5 greatly alleviated liver injury and improved survival in mice, suggesting that CCL5 blockage might be a promising therapeutic strategy for the treatment of DILI patients [176]. Pharmacological inhibition of CCL2 suppressed the migration of Ly6C^+^ monocytes and accelerated regression of liver fibrosis in mice with both CCl_4_ challenge and methionine-choline-deficient diet treatment [177]. Cenicriviroc, an oral inhibitor of CCR2 and CCR5, significantly reduced the recruitment of hepatic Ly6C^+^ monocytes, inhibited alcohol-induced steatohepatitis and ameliorated liver fibrosis in mice [177,178]. A randomized, controlled clinical trial has revealed that cenicriviroc treatment is well-tolerated and achieves anti-fibrotic benefits in patients with nonalcoholic steatohepatitis, particularly in those with advanced fibrosis [179,180].

B cells have been suggested to play important roles in fibrogenesis. B cell depletion therapy by rituximab is well tolerated and has achieved clinical improvements in SSc patients [127,128,181]. Rituximab treatment led to a decrease in disease activity index and disease severity index. Moreover, IL-6 levels were decreased during the follow up [128]. Mechanistically, it has been shown that rituximab-mediated B cell depletion improves skin fibrosis regression through regulating TGFβ-Dkk-1 axis in SSc patients [127]. Moreover, treatment with rituximab may serve as an effective, potentially life-saving, therapeutic intervention for patients with severe interstitial lung disease associated with connective tissue disease [182]. Up to date, several clinical trials have been initiated to investigate the safety and efficacy of rituximab in combination with other therapies in treating IPF patients (NCT03584802, NCT01969409, and NCT03286556).

Immune cell targeted therapies for fibrotic diseases have been developing rapidly during recent years. Preclinical studies in fibrotic animal models have provided important information on novel immune-based therapeutic strategies by targeting different immune cells. Moreover, the molecular insights into fibrogenesis have suggested clinical therapeutic potential by targeting key molecular components, including PI3K/mTOR and JAK-STAT pathways [183,184,185,186]. Future controlled clinical trials are essential to evaluate the safety and efficacy of potential therapies in patients with fibrotic diseases.

## 6. Conclusions and Future Perspectives

**Figure 1:**
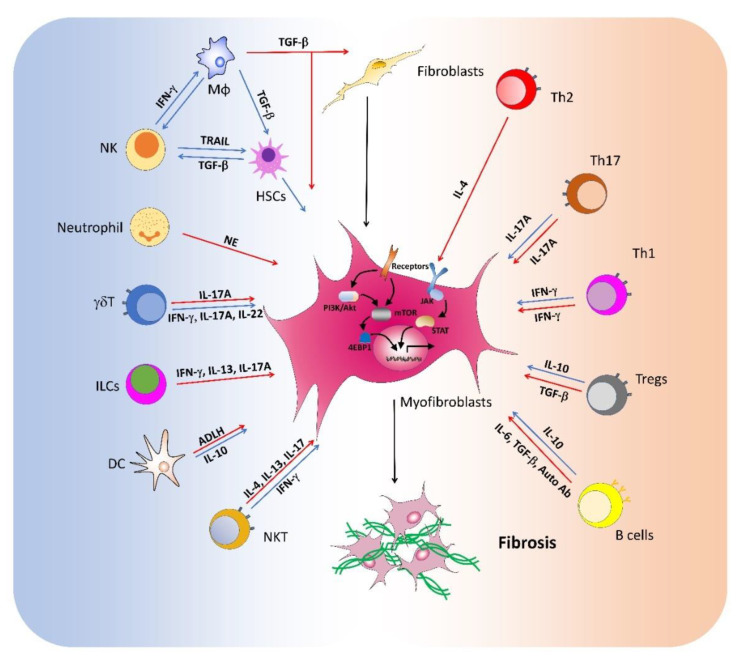
Roles of immune cells in the pathogenesis of fibrosis. During inflammatory responses, many immune cell populations with diverse functions are activated to produce multiple cytokines that either activate (red arrow) or suppress (blue arrow) the differentiation, proliferation, and collagen production of myofibroblasts, promoting or suppressing the development of fibrosis in various diseases.

Fibrosis is a common pathway to organ injury and failure in a variety of diseases. Many immune cell populations are involved in the pathogenesis of fibrosis with diverse functions (Figure 1, Table 1). The recruitment and activation of both innate and adaptive immune cells orchestrate the fibrotic process. The interactions between immune cells and myofibroblast are key events in fibrogenic responses. Activated immune cells produce multiple cytokines that modulate the differentiation, proliferation, survival, and collagen production of myofibroblasts. Moreover, macrophages and other cells also secrete massive amounts of TGF-β that directly contributes to fibrosis. Current studies have suggested multifaceted functions of immune cells in fibrotic diseases, possibly due to dynamic changes in microenvironment during disease development. Moreover, most immune cell types are heterogeneous with functional plasticity modulated by both systemic and microenvironmental factors. Thus, the cellular identities and local niches are of key significance for their functions in fibrosis. The functions of both immune cells and fibroblasts are tightly regulated by molecular network. The perturbed mTOR and JAK-STAT signaling pathways contribute to immune dysregulation and subsequent fibrosis development. Multiple cytokines and growth factors activate these critical molecular mediators in immune cells and fibroblasts, which exert diverse functions during fibrosis. Further studies on the regulation and functions of immune cells in fibrosis will facilitate the development of immune cell-based therapies for the treatment of fibrotic diseases.

## Figures and Tables

**Table 1:** The roles of immune cells in the development fibrosis.

Cell Subsets	Roles in Fibrosis	Diseases/Models	References
Macrophages	Promote liver fibrosis	NFκB-induced or CCl_4_-induced liver fibrosis BDL-induced liver fibrosis	[8,9,10,11]
	M2 macrophages promote renal fibrosis	UUO-induced renal fibrosis	[16]
	Kupffer cells promote fibrosis	CCl_4_-induced liver fibrosis	[4,21]
	Kupffer cells contribute to liver fibrosis resolution	Thioacetamide-induced liver fibrosis	[22]
	Segregated nucleus-containing atypical monocytes or alveolar macrophages promote lung fibrosis	IL-10 or bleomycin-induced pulmonary fibrosis	[17,18,19,20]
	M1 cells express MMP12 to degrade ECM	CCl_4_-induced liver fibrosis	[15]
Neutrophils	Promote lung fibrosis	Experimental hypersensitivity pneumonitis Bleomycin-induced pulmonary fibrosis	[30,31]
	NE promote lung fibrosis	Asbestos-induced lung fibrosis	[32,33]
NK cells	Suppress liver fibrosis	CCl_4_-induced and HBV associated liver fibrosis	[35,36,37,38]
	Suppress cardiac fibrosis	Autoimmune myocarditis	[39]
	Promote liver fibrosis	Low-dose rotavirus infection	[40]
	Promote renal fibrosis	Injury-induced renal fibrosis	[41]
ILC1	Promote adipose fibrosis	High-fat diet-fed obese mice	[48]
ILC2	Promote lung fibrosis	Bleomycin-induced pulmonary fibrosis	[45,47]
ILC3	Promote liver fibrosis	CCl_4_-induced liver fibrosis	[44,49]
γδT cells	Suppress liver fibrosis	CCl_4_-induced liver fibrosis	[50]
	Suppress lung fibrosis	*B. subtilis*-induced pulmonary fibrosis Bleomycin-induced pulmonary fibrosis	[51,52,53]
	Promote liver fibrosis	*S. japonicum* -induced liver fibrosis	[54]
Dendritic cells	Suppress lung fibrosis	Mice exposed to adenoviral gene transfer of TGF-β1	[59]
	IL-10-producing DCs suppress heart inflammation and fibrosis	Chronic Chagas disease cardiomyopathy model	[62]
	pDCs suppress lung and skin fibrosis	Bleomycin-induced SSc model	[64]
	CD11b^+^ DCs promote ocular mucosal fibrosis	Allergic eye disease model	[65]
NKT cells/ MAIT cells	NKT cells promote liver fibrosis	CCl_4_-treated HBV-transgenic mice	[66]
	CD1d-dependent NKT cells suppress kidney fibrosis	Diet-induced NAFLD mouse model	[67]
	iNKT cells suppress lung fibrosis	Bleomycin-induced pulmonary fibrosis	[69]
	invariant NKT cells promote liver injury and fibrosis	Autoimmune cholangitis mouse model, mice fed with high fat diet	[71,72]
	MAIT cells promote liver fibrosis	CCl_4_-induced or bile duct ligation-induced liver fibrosis	[76]
Th1 cells	Suppress lung fibrosis	Bleomycin-induced pulmonary fibrosis	[79,80]
	Promote peritoneal fibrosis	*S. epidermidis-*induced peritoneal fibrosis	[81]
	Promote cardiac fibrosis	Thoracic aortic constriction-induced cardiac fibrosis	[82]
Th2 cells	Promote liver fibrosis	Bacterial infection-associated hepatic fibrosis	[84]
	Promote airway fibrosis	House dust mite -induced allergic disorders	[85]
Th17 cells	Promoted skin and lung fibrosis	Bleomycin-induced SSc model	[87]
	Promote lung fibrosis	Bleomycin-induced pulmonary fibrosis	[86]
	Suppress lung fibrosis	*B. subtilis*-induced pulmonary fibrosis	[51]
Tregs	Suppress lung fibrosis	Bleomycin-induced pulmonary fibrosis (late stage)	[98]
	Suppress liver fibrosis	BDL-induced liver fibrosis	[101]
	Suppress skin fibrosis	Bleomycin-induced SSc model	[102]
	Suppress kidney fibrosis	Injury-induced renal fibrosis	[103]
Tfh cells	Promote dermal fibrosis	GVHD-SSc model	[115]
	Promote lung fibrosis	Bleomycin-induced pulmonary fibrosis	[117]
B cells	Promote lung fibrosis	Bleomycin-induced pulmonary fibrosis	[119,120]
	Promote skin fibrosis	TSK/+ mice	[121,122]
	Promote liver fibrosis	CCl_4_-induced liver fibrosis	[123]
	IL-6-producing B cells promote skin and lung fibrosis	Bleomycin-induced SSc model	[124]
	Bregs suppress skin and lung fibrosis	Bleomycin-induced SSc model	[124]
	Bregs promote lung fibrosis	Silica-induced pulmonary fibrosis	[129]

## Abbreviations

α-SMA	α-smooth muscle actin
α-Galcer	α-Galactosylceramide
AhR	aryl hydrocarbon receptor
AILD	autoimmune liver diseases
ASK1	apoptosis signal-regulating kinase 1
BAFF	B-cell activating factor
BALF	bronchoalveolar lavage fluid
BDL	Bile duct ligation
Bregs	regulatory B cells
CCl_4_	carbon tetrachloride
CCL2	C-C motif chemokine ligand 2
CKD	chronic kidney disease
COPD	chronic obstructive pulmonary disease
DC	Dendritic cell
DILI	drug-induced liver injury
ECM	extracellular matrix
FGF-9	fibroblast growth factor 9
GVHD	graft-versus-host disease
NASH	nonalcoholic steatohepatitis
HBV	hepatitis B virus
HCV	hepatitis C virus
HMF	hepatic myofibroblast
HSCs	hepatic stellate cells
ICOS	inducible co-stimulator
ILCs	innate lymphoid cells
iNKT cells	invariant natural killer T cells
IPF	idiopathic pulmonary fibrosis
JAK	Janus kinase
MAIT cells	mucosal-associated invariant T cells
MAPK	mitogen-activated protein kinase
MMPs	matrix metalloproteinases
mTOR	mammalian target of rapamycin
NAFLD	nonalcoholic fatty liver disease
NASH	nonalcoholic steatohepatitis
NE	neutrophil elastase
NK cells	natural killer cells
NKT cells	natural killer T cells
PBC	primary biliary cholangitis
PDGF	platelet-derived growth factor
PTECs	proximal tubular epithelial cells
SSc	systemic sclerosis
STAT	signal transducers and activators of transcription
Tfh cells	T follicular helper cells
TGF-β	transforming growth factor-β
TRAIL	TNF-related apoptosis-inducing ligand
Tregs	regulatory T cells
UUO	unilateral ureteral obstruction
